# Association between Diet Quality and Risk of Type 2 Diabetes Mellitus in Patients with Coronary Heart Disease: Findings from the CORDIOPREV Study

**DOI:** 10.3390/nu16081249

**Published:** 2024-04-22

**Authors:** Lorenzo Rivas-Garcia, Gracia M. Quintana-Navarro, Juan F. Alcala-Díaz, Jose D. Torres-Peña, Antonio P. Arenas-de Larriva, Oriol Alberto Rangel-Zuñiga, Alejandro López-Moreno, Maria M. Malagon, Niki Katsiki, Pablo Perez-Martinez, Jose Lopez-Miranda, Javier Delgado-Lista

**Affiliations:** 1 Lipids and Atherosclerosis Unit, Department of Internal Medicine, Reina Sofia University Hospital, 14004 Córdoba, Spain; 2 Maimónides Biomedical Research Institute of Córdoba (IMIBIC), 14004 Córdoba, Spain; 3 CIBER Fisiopatologia de la Obesidad y la Nutricion (CIBEROBN), Instituto de Salud Carlos III, 28029 Madrid, Spain; 4 Department of Medical and Surgical Sciences, University of Córdoba, 14004 Córdoba, Spain; 5 Department of Cell Biology, Physiology and Immunology, University of Córdoba, 14004 Córdoba, Spain; 6 Department of Nutritional Sciences and Dietetics, International Hellenic University, 57400 Thessaloniki, Greece; 7 School of Medicine, European University Cyprus, Nicosia 2404, Cyprus

**Keywords:** diet quality, nutrient density, NRF9.3, coronary heart disease

## Abstract

The incidence of type 2 diabetes mellitus (T2DM) is growing in Western countries. Nutritional interventions that promote high-quality dietary patterns could help reverse this trend. We aimed to evaluate whether changes in Nutrient-Rich Food Index 9.3 (NRF9.3) were related to the risk of developing T2DM in patients with coronary heart disease (CHD). The study was carried out in the context of two healthy dietary interventions (a Mediterranean and a low-fat diet). For this purpose, we evaluated all the patients in the CORDIOPREV study without T2DM at baseline. Data were obtained during the first 5 years of dietary intervention. The score was calculated using the Food Frequency Questionnaires at baseline and after 1 year of intervention. After 5 years of follow-up, 106 patients developed T2DM (incident-T2DM), while 316 subjects did not (non-T2DM). Total NRF9.3 score and changes during the first year of intervention were compared between incident-T2DM and non-T2DM. Incident-T2DM showed less improvement in NRF9.3 than non-T2DM (*p* = 0.010). In the multi-adjusted Cox proportional hazard study, patients with greater improvement in NRF9.3 had over 50% less risk of developing T2DM compared with the lowest tertile (HR 2.10, 95%, CI = 1.12–3.56). In conclusion, improved diet quality in terms of nutrient density after the dietary intervention was associated with a lower risk of T2DM in patients with CHD.

## 1. Introduction

Type 2 diabetes mellitus (T2DM) is a chronic disease mediated by an abnormal carbohydrate metabolism, causing multiple impairments in several organs and systems [1]. The World Health Organization (WHO) confirmed that T2DM incidence is growing and that its prevalence has doubled since 2014 [2]. T2DM is closely associated with overweight, obesity and consumption of unhealthy diets [3,4]. In contrast, adherence to certain high-quality dietary patterns has revealed positive effects in preventing its incidence. In this context, an inverse linear association between adherence to the Mediterranean diet and T2DM development has been observed across different prospective cohort studies [5,6]. Of note, the Mediterranean diet also plays an important role in the prevention and management of metabolic syndrome [7,8] and non-alcoholic fatty liver disease [9], which are both linked to T2DM incidence. Other dietary patterns that emphasize nutrient-dense plant-based foods, such as the Dietary Approaches to Stop Hypertension (DASH) [10] and some vegetarian diets [11], have also been shown to be associated with lower T2DM risk in general populations. The potential favorable effects of these diets on T2DM prevention have been attributed to the intake of certain food groups such as fruit and vegetables, legumes, whole grains and nuts, in preference to processed products and sugar-sweetened beverages, providing a sufficient quantity of nutrients but with a low energy content, since not only the total energy is important, but also the nutritional value of the overall diet [12].

The concept known as the nutrient density of a diet indicates the ratio between nutrients and total energy intake, and has been identified as a good indicator of diet quality [13,14]. Over the last few years, a few diet quality indices (DQIs) have been proposed. These scales are mostly based on the macronutrient intake, without taking into account the dietary composition of the micronutrients, and are mainly used in public health and nutritional epidemiology to categorize individuals according to the adequacy of their dietary habits [15,16]. Among these, the Alternative Healthy Eating Index 2010 (AHEI-2010) is currently one of the most widely-used DQIs since it is not only based on the current Dietary Guidelines for Americans but also includes certain nutrients and foods associated with the development of chronic diseases [17]. The level of the AHEI-2010 index was reported to predict the risk of cardiovascular disease, T2DM, and mortality from cardiovascular disease and cancer in several prospective studies [17,18,19].

Recently, a new DQI has been suggested, the Nutrient-Rich Food Index 9.3 (NRF9.3), as a promising tool not only to measure the nutrient density of individual foods, meals or total diets, but also to establish an association with the incidence and development of certain diseases [20,21]. The NRF9.3 index is based on recommended daily values (RDV) of nutrients whose consumption should be encouraged [22], comprising fiber and proteins, as macronutrients, as well as several micronutrients involved in multiple physiological functions. These include calcium, a mineral that strengthens bone structure [23], minerals with antioxidant properties like magnesium [24], vitamin E and vitamin C [25], and other nutrients associated with pathways related to cellular metabolisms such as iron, which is involved in the erythrocyte metabolism and immunity system, vitamin A, associated with several processes of cell maintenance [26] and potassium, a mineral related to the regulation of cellular membranes. Moreover, the NRF9.3 also includes maximum recommended daily values (MRDV) of certain nutrients whose intake should be limited, such as saturated fats, sodium and added sugars, that are also found to be associated with the ultra-processed food industry and are directly related to the pathophysiology of obesity, cardiovascular disease and T2DM [27]. Certain patient populations require the prescription of a healthy diet in the long term. Therefore, it could be of interest to identify a tool that can assess the expected efficacy of such an intervention.

Considering all the above, the present study aimed to evaluate whether the quality of diet evaluated by the NRF9.3 index is associated with the risk of developing T2DM in patients with coronary heart disease (CHD). Moreover, we studied whether NRF9.3 was an efficient tool to predict T2DM incidence following a dietary intervention. If so, it would point to the potential use of this scale not only as suitable DQIs, but also as potential nutritional markers of T2DM risk.

## 2. Materials and Methods

### 2.1. Study Subjects

This work was carried out within the context of the CORDIOPREV study (Clinicaltrials.gov NCT00924937). It is a randomized, controlled trial including 1002 patients with CHD, who followed one of two healthy diets (a Mediterranean diet and a low-fat diet) for 7 years [28]. From November 2009 to February 2012, the patients were recruited mainly at the Reina Sofia University Hospital (Cordoba, Spain), and other hospitals located in the provinces of Cordoba and Jaen (Spain). Inclusion and exclusion criteria have been previously detailed [28]. Briefly, patients aged between 20 and 75 years, with established CHD but without clinical events during the last 6 months, were willing to follow a long-term monitoring study, and had no other serious illnesses. No intervention to increase physical activity was included. The patients consented to participate in the study. The local ethics committees approved the trial protocol and amendments, according to the Helsinki Declaration and good clinical practices. The results of the main objective of the CORDIOPREV study have been published [29].

All patients in the CORDIOPREV study without T2DM, according to the American Diabetes Association (ADA) diagnosis criteria [30] (i.e., fasting plasma glucose ≥ 126 mg/dL, 2-h plasma glucose in the 75 gr oral glucose tolerance test (OGTT) ≥ 200 mg/dL or plasma glycated hemoglobin (HbA1c) levels ≥ 6.5%) at the beginning of the study (n = 462), were initially included. Of these patients, 40 patients were excluded from the present analysis: 16 died, 8 discontinued the study, 14 did not provide dietary data at baseline or during follow-up and 2 patients had extreme baseline values for total energy intake: <500 kcal/day or >3500 kcal/day for women and <800 kcal/day or >4000 kcal/day for men, according to the established criteria proposed by Willet et al. [31]. Therefore, a total of 422 patients were finally included in this study. Of these, 106 developed T2DM after 5 years (incident-T2DM group), whereas the remaining 316 did not (non-T2DM group). Supplementary Figure S1 describes the participant flow chart. Table 1 summarizes the baseline patient characteristics.

### 2.2. Dietary Intake Assessment

We included dietary data from baseline and at the 1-year follow-up visit. Dietary intake was assessed via a validated 137-item semi-quantitative food-frequency questionnaire (FFQ) [32]. Trained dietitians administered the FFQ in a face-to-face interview in which participants reported how often, on average, they had consumed standard portions of each food item over the previous year. Reported frequencies of consumption of each food item were transformed into daily intakes and multiplied by the weight of the standard portion size to obtain the intake in grams per day.

Energy and nutrient intake were calculated by applying the Spanish food composition tables [33,34] to daily food intake. Added sugar consumption was estimated using the standardized 10-step method described by Louie et al. (Supplementary Figures S2 and S3) [35].

### 2.3. Nutrient-Rich Food Index 9.3 Calculation

The nutrient density of the total diet was assessed using the NRF9.3 score [22]. This validated DQI is based on 12 nutrients: 9 nutrients to encourage (protein, fiber, vitamin A, vitamin C, vitamin E, calcium, iron, potassium, and magnesium) and 3 nutrients to limit (saturated fat, added sugars, and sodium). The NRF9.3 score was calculated as described by Ruiz et al. [36]. First, the daily intake of each nutrient was adjusted for 2000 kcal and expressed as a percentage of the reference daily value. Next, the NRF9.3 score for each patient was calculated as follows:

$$\mathrm{NRF}9.3=\left(\sum_{i=1}^{9}\frac{\mathrm{Intake}\;i\;/\;\mathrm{Energy}\times 2000}{\mathrm{RDVi}}\times 100\right)\;-\left(\sum_{j=1}^{3}\frac{\mathrm{Intake}\;j\;/\;\mathrm{Energy}\;\times 2000}{\mathrm{MRDVj}}\times 100\right)$$

where Intake i is the daily intake of each nutrient i to encourage; Intake j is the daily intake of each nutrient j to limit; Energy is daily energy intake; RDVi is the recommended daily value for nutrients i and MRDVj is the maximum recommended daily value for nutrients j.

For each of the 12 nutrients, each percentage of the reference daily value was capped at 100. For nutrients to limit, a value of 0 was assigned for daily intakes below the MRDV.

The maximum possible score was 900, reflecting a diet where the intake per 2000 kcal for nutrients to encourage was above the reference daily value and the intake of nutrients to limit was below the reference daily value [36].

The NRF9.3 was calculated using reference daily values according to Regulation (EU) No.1169/2011 of the European Parliament [37], except for fiber and added sugars, for which the European Food Safety Authority (EFSA) [38] and the WHO [39] recommendations were used, respectively. The RDVs for nutrients to encourage were: 50 g for protein, 25 g for fiber, 800 µg RAE for vitamin A, 80 mg for vitamin C, 12 mg for vitamin E, 800 mg for calcium, 14 mg for iron, 375 mg for magnesium, and 2000 mg for potassium. MRDV for nutrients to limit were: 20 g for saturated fat, 50 g for added sugars, and 2400 mg for sodium. All these parameters were calculated according to the methodology proposed previously [36].

The NRF9.3 score was evaluated both at baseline and after 1 year of dietary intervention. To evaluate the changes occurring in time, we also calculated the Δchanges (ΔNRF9.3 = changes between baseline and the end of the first year of intervention).

### 2.4. Alternative Healthy Eating Index-2010 Calculation

Dietary quality was also assessed using the AHEI-2010, which is based on 11 dietary factors that are predictive of chronic diseases [17]. The AHEI-2010 consists of 6 components that should be consumed in adequate amounts (i.e., vegetables, fruit, whole grains, nuts and legumes, long-chain omega-3 fats, and other polyunsaturated fatty acids), 1 component that should be consumed in moderation (i.e., alcohol intake), and 4 components to be avoided (i.e., sugar-sweetened beverages, red/processed meats, sodium, and trans fats). Each of the 11 components is scored from 0 (minimal score) to 10 (maximal score), with intermediate values scored proportionally, as described by Chiuve et al. [17]. (Supplementary Table S1). The total AHEI-2010 score is obtained by adding up all the component scores, and ranges from 0 (low-quality diet) to 110 (high-quality diet).

For the present analysis, the total AHEI-2010 score was calculated both at baseline and after 1 year of dietary intervention. To evaluate the changes occurring in time, we also calculated the Δchanges (ΔAHEI2010 = changes between baseline and the end of the first year of intervention).

### 2.5. Anthropometric Measurements and Laboratory Tests

Venous blood was collected in EDTA tubes. Some variables such as anthropometric, lipid variables, serum insulin and plasma glucose have been reported previously [40]. Moreover, other measures including insulin sensitivity index (ISI), homeostatic model assessment of insulin resistance (HOMA-IR), insulinogenic index (IGI) and disposition index were calculated as previously described [41].

### 2.6. Statistical Analysis

All the analyses were performed using the Statistical Package for Social Science 20.0 (SPSS, Chicago, IL, USA). The data are represented as the mean ± standard error of the mean (SEM) for continuous variables and as proportions for categorical variables. The normal distribution of the quantitative variables was evaluated using the Kolmogorov–Smirnov test, and the comparison of qualitative variables was performed using the Chi-square test. The changes between the groups for continuous variables were compared using an unpaired *t*-test or univariate ANOVA.

To assess the differences between incident-T2DM and non-T2DM groups in relation to ΔNRF9.3 an ANOVA test was conducted.

A Cox proportional hazards regression analysis was carried out to measure the probability of developing T2DM according to the tertiles of basal NRF9.3 and ΔNRF9.3. All analyses were adjusted for age, sex, statin treatment, smoking habits, body mass index (BMI), low-density lipoprotein cholesterol (LDL-C), high-density lipoprotein cholesterol (HDL-C), triglycerides (TG), HOMA-IR, disposition index (DI), ISI and IGI. Sensitivity tests were also performed to rule out any differences in the outputs when excluding the patients who developed T2DM during the first year of intervention. The results are shown in the Supplementary Figures S4 and S5. Bonferroni’s method was used for correcting multiple tests. Differences were considered significant when *p* (2-sided) was <0.05.

## 3. Results

### 3.1. Baseline Patient Characteristics

The baseline characteristics of the patients included in this study (incident-T2DM and non-T2DM groups) are shown in Table 1. BMI, waist circumference, HOMA-IR, HbA1c, fasting insulin and glucose levels were significantly higher in incident-T2DM patients compared with non-T2DM patients (all *p* < 0.05). In contrast, TG, DI, IGI and ISI were lower in incident-T2DM patients compared with non-T2DM patients (all *p* < 0.05). For the rest of the parameters studied, there were no significant differences between the 2 groups. We also observed no differences in the baseline DQI scores between incident-T2DM and non-T2DM patients. Additionally, it is remarkable that there were fewer women than men in the study, although the differences in distribution in both groups were similar.

### 3.2. Effect of the Dietary Intervention on NRF9.3 Scores

**Figure 1:**
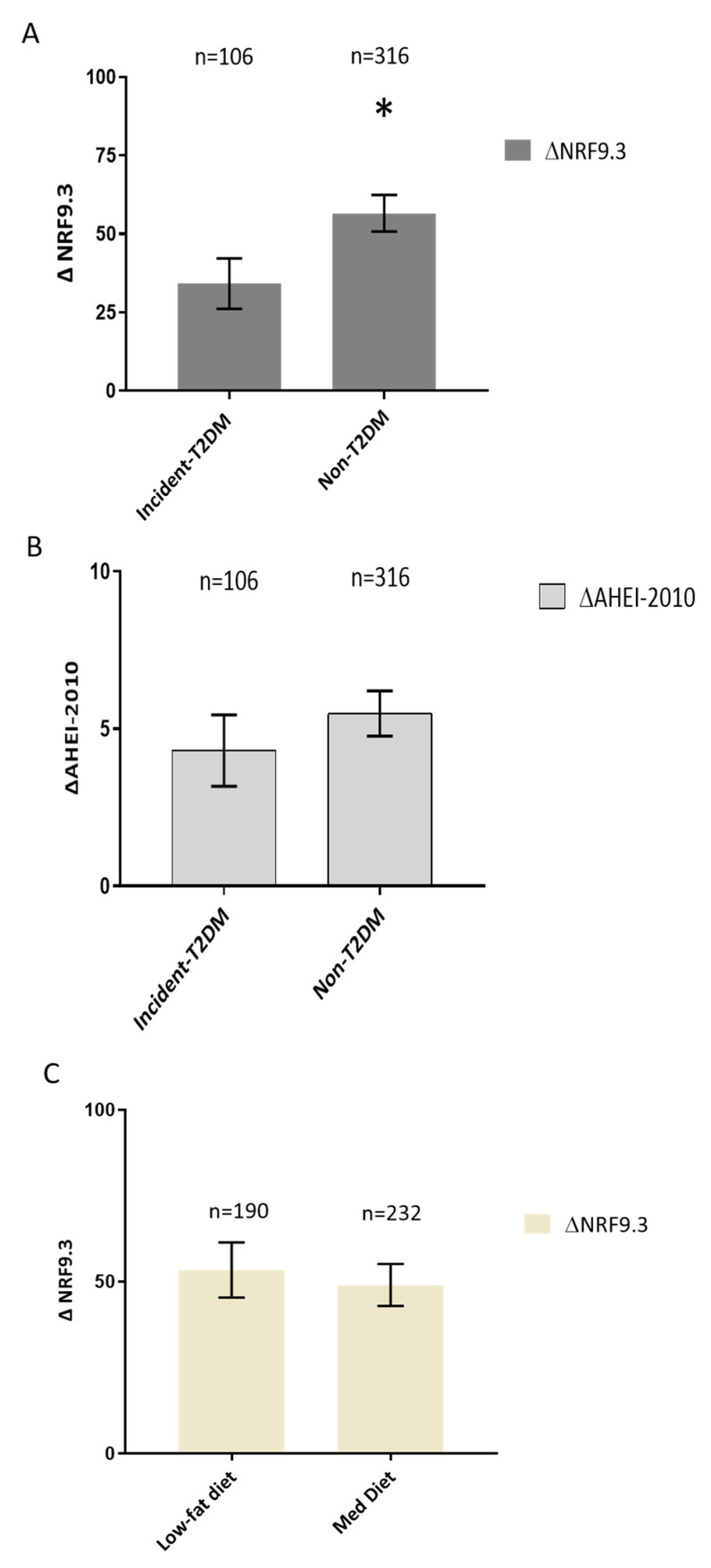
(**A**) Effect of the dietary intervention on NRF9.3 score and diabetes status. Data are presented as Δchanges of NRF9.3 produced between post- and pre-intervention ± SEM. Variables were compared using the analysis of variance (univariate ANOVA) adjusted by age, sex, statin treatment, smoking habits, BMI, LDL, HDL, TG, HOMA-IR, ISI, DI and IGI. Incident-T2DM (n = 106) and Non-T2DM (n = 316). Differences were considered to be significant when *p* < 0.05. * Significant differences between incident-T2DM and Non-T2DM. (**B**) Effect of the dietary intervention on AHEI-2010 score and diabetes status. Data are presented as Δchanges of AHEI-2010 produced between post- and pre-intervention ± SEM. Variables were compared using the analysis of variance (univariate ANOVA) adjusted by age, sex, statin treatment, smoking habits, BMI, LDL, HDL, TG, HOMA-IR, ISI, DI and IGI. Incident-T2DM (n = 106) and Non-T2DM (n = 316). Differences were considered to be significant when *p* < 0.05. (**C**) Effect of the dietary intervention on NRF9.3 score according to randomized diet group. Data are presented as Δchanges of NRF9.3 produced between post- and pre-intervention ± SEM. Variables were compared using the analysis of variance (univariate ANOVA) adjusted by age, sex, statin treatment, smoking habits, BMI, LDL, HDL, TG, HOMA-IR, ISI, DI and IGI. Low-fat diet (n = 190) and Mediterranean diet (n = 232). Differences were considered to be significant when *p* < 0.05. Abbreviation: NRF9.3, Nutrient-Rich Food index 9.3AHEI-2010, Alternative Healthy Eating Index-2010; BMI, Body mass index; LDL, Low-density lipoprotein; HDL, high-density lipoprotein; TG, tryglicerides; HOMA-IR, homeostatic model assessment; ISI, insulin sensitivity index; DI, disposition index; IGI, insulinogenic index.

Figure 1 shows that incident-T2DM patients exhibited lower values of the ΔNRF9.3 score compared with non-T2DM patients (*p* = 0.010). Additionally, we assessed whether the type of intervention influenced the improvement in the NRF9.3 parameter. In this case, there were no significant differences between the two types of dietary intervention (Figure 1B).

### 3.3. Effect of the Dietary Intervention on AHEI-2010 Scores

No significant differences were observed in ΔAHEI-2010 score between incident-T2DM and non-T2DM patients (Figure 1C).

### 3.4. Analysis of the Probability of Type 2 Diabetes Mellitus Incidence

**Figure 2:**
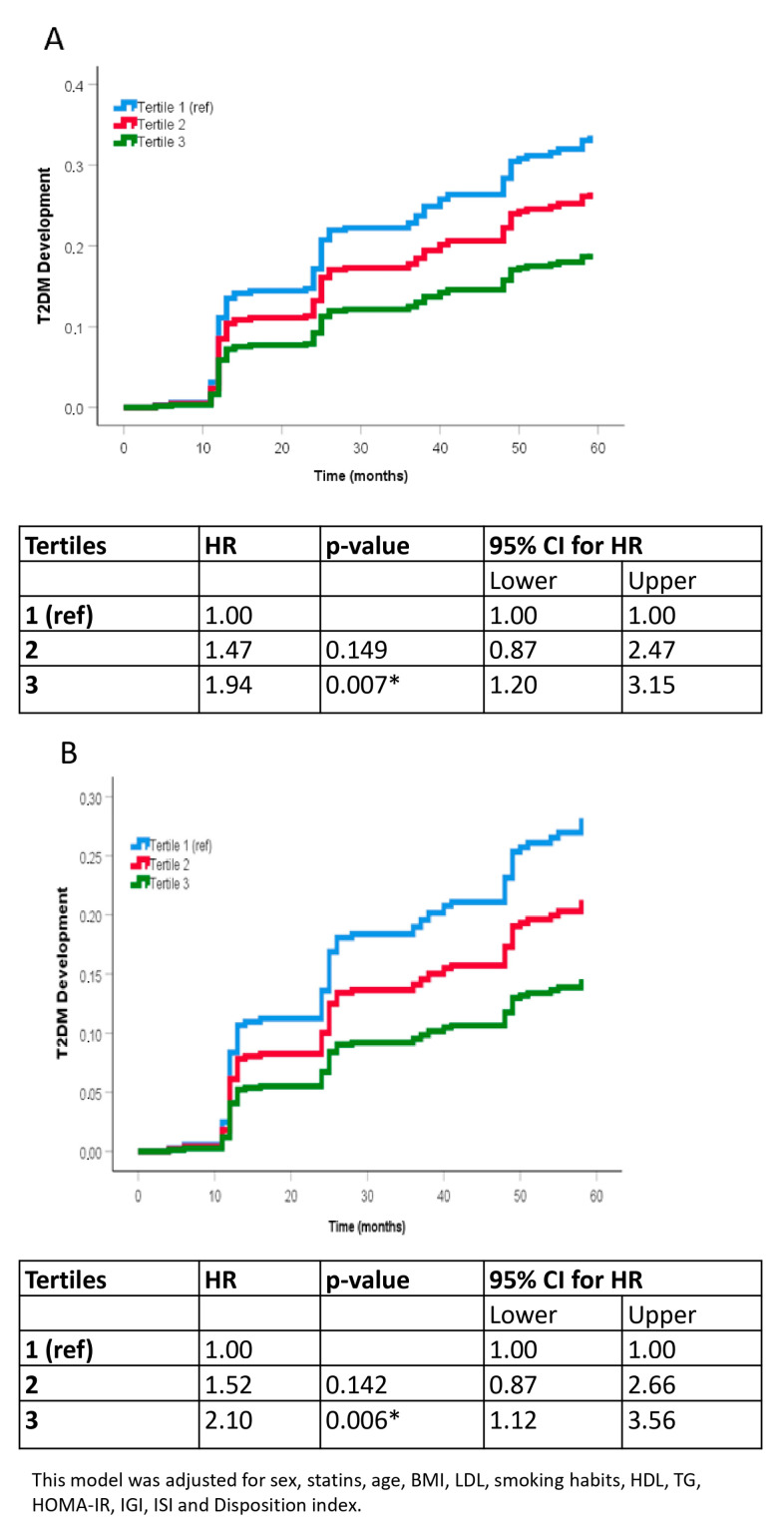
Probability of T2DM development by COX analysis according to the tertiles of ΔNRF9.3. (**A**) unadjusted model. (**B**) adjusted model controlled for sex, statins, age, BMI, LDL, smoking habits, HDL, TG. C fully adjusted model controlled for sex, statins, age, BMI, LDL, smoking habits, HDL, TG., HOMA-IR, ISI, DI and IGI. Reference was the Tertile 1 (lowest). The hazard ratio (HR) between groups was calculated. Abbreviation: BMI, Body mass index; LDL, Low-density lipoprotein; HDL, high-density lipoprotein; TG, Tryglicerides, HOMA-IR, homeostatic model assessment; ISI, insulin sensitivity index; DI, disposition index; IGI, insulinogenic index. * *p* < 0.05.

We divided the patients into tertiles according to their ΔNRF9.3 score and performed a COX proportional hazards regression analysis after a median follow-up of 5 years to evaluate the risk of T2DM incidence. Patients who exhibited a greater improvement in NRF9.3 (tertile 3) showed a significantly lower probability of developing T2DM than those patients with the lowest ΔNRF9.3 score (tertile 1) (unadjusted HR of 1.94 tertile 1 vs. tertile 3, Figure 2A; and HR of 2.10 after adjusting for age, sex, statin therapy, BMI, LDL-C, smoking habits, HDL-C, TG, HOMA-IR, DI, IGI and ISI, Figure 2B).

### 3.5. Sensitivity Analyses

We repeated all statistical tests after excluding patients who developed T2DM during the first year of intervention. The obtained results were similar to those obtained for the total population (Supplementary Figures S4 and S5).

## 4. Discussion

In the present study, after one year of consumption of two healthy diets, there were similar improvements in the NRF9.3 score (which indicates a high intake of beneficial nutrients and/or a low consumption of deleterious nutrients relative to total energy intake), that were related to a lower probability of incident T2DM in the long-term (5 years). Furthermore, the extent of change (increase) in NRF9.3 score at the end of the first year of intervention was inversely associated with the risk of developing T2DM; patients in the lowest tertile of ΔNRF9.3 had a greater risk for T2DM incidence compared with those in the highest tertile (HR 2.10). In contrast, changes in the AHEI-2010 were not associated with the incidence of T2DM at 5 years.

Lifestyle interventions and, in particular, consumption of healthy dietary patterns, are widely recognized to be effective in reducing the risk of developing T2DM. In this context, different studies based on dietary recommendations to decrease the intake of total and saturated fat, increase the consumption of fiber and implement regular physical activity have shown a reduction in the incidence of metabolic syndrome and T2DM in subjects with impaired glucose tolerance [42,43]. Of note, we recently demonstrated that dietary intervention with two healthy dietary patterns (a Mediterranean diet and a low-fat diet) exhibited the same benefits in decreasing the cases of T2DM onset in prediabetic patients with CHD [44].

The present study evaluated diet quality in terms of nutrient density. To the best of our knowledge, no previous dietary clinical study has analyzed the association between the NRF9.3 diet quality index and the risk of T2DM development. In fact, the only clinical association previously with this DQI was the overall survival of ovarian cancer patients [45]. Therefore, NRF9.3 could serve as a new tool for monitoring other conditions such as obesity or inflammation. NRF9.3 is a nutrient profiling method based on nutrient density rather than absolute intake of foods/nutrients [21,46] and could provide a better and more useful tool for identifying dietary patterns that provide most of the nutrients in the correct proportions. An improvement in the NRF9.3 score was observed in the patients in our study, regardless of the dietary intervention group (Mediterranean diet or low-fat diet). These findings extend the results of a previous work, in which we demonstrated that a high-intensity dietary intervention with two healthy diets improved diet quality and that this improvement persisted during the 7 years of follow-up [14]. The inverse relationship between the NRF9.3 and the risk of developing T2DM found in the present study suggests that patients who changed their dietary habits early (within 1 year) towards a healthy, nutrient-dense diet (a Mediterranean diet or a low-fat diet) and did so more efficiently during the follow-up of the study, were less likely to develop T2DM in the following years. This could be attributed to the effects of some nutrients that comprise the NRF9.3 in glucose metabolism. For example, the intake of dietary magnesium has been reported to lower insulin resistance markers such as the HOMA-IR and HOMA-β [47]. In addition, magnesium plays a role in the process of insulin secretion and signaling [48], and, specifically, mediates phosphorylation of the insulin receptor and other downstream signal kinases of the target cells [49]. Other nutrients, such as vitamin C and vitamin E have shown antidiabetic properties based on their antioxidant activities, reducing and/or modulating oxidative damage [50,51,52]. Moreover, one of the main characteristics of NRF9.3 is the consideration of nutrients (saturated fats, sodium, and added sugars) which are found in ultra-processed foods, and their consumption is closely associated with chronic cardiometabolic diseases [53]. Therefore, apart from the nutrients and substances included in this index, we may also record part of the patient’s behavior in terms of food consumption [53].

Our results demonstrate that NRF9.3 can more effectively predict the risk of developing T2DM compared to other DQIs. In fact, we did not report differences between Incident T2DM and non-T2DM regarding the improvement of AHEI-2010. These results agreed with previous research. In that way, some authors demonstrated that changes in AHEI-2010 following a year-long dietary intervention were not correlated with the incidence of T2DM over a three-year follow-up period among participants in the Diabetes Prevention Program (DPP) [4]. Moreover, the results obtained in our study also coincide with a study conducted in the Atherosclerosis Risk in Communities (ARIC) cohort, where AHEI-2010 scores improved slightly over 6 years, but there was no significant association between changes in AHEI-2010 and risk of T2DM [54]. In contrast with our results, Ley et al. found that an improvement in AHEI-2010 scores over 4 years was related to a lower T2DM risk in the subsequent 4 years in three large cohorts of U.S. health professionals [55]. However, this study was conducted in a predominantly female population, who were younger, and at a lower risk of developing T2DM than the patients included in our study. The incidence rate of T2DM was also much lower in the U.S. health professionals pooled study population than in our study (5 vs. 58.1 cases/1000 person-years, respectively) [56].

This disparity may stem from differences between NRF9.3 and AHEI-2010 in terms of the number and types of dietary components assessed, optimal cut-off values, and scoring ranges. Notably, NRF9.3 directly evaluates the intake of dietary fiber and added sugars, whereas AHEI-2010 measures these indirectly. Given the established associations of these nutrients with T2DM development [57], a DQI that more accurately quantifies them may exhibit a stronger link with incident T2DM.

Moreover, the NRF9.3 presents a very wide score range compared with the AHEI-2010, and, thus has a greater capacity to reflect smaller changes in diet quality. This is of special interest in populations at high risk of developing T2DM, in which small changes in diet quality could have a great impact on the prevention of the disease. Furthermore, we included variables closely related to T2DM for building the models, to control their actions as confounding factors. HOMA-IR and DI were included as parameters for evaluating the beta-cell function, which is directly associated with the risk of developing T2DM [58]. Moreover, other parameters such as IGI and ISI were included to build the models. Thus, our results support the positive effects of healthy dietary patterns in preventing the development of T2DM. Some of the previous studies did not include these variables in their models [4,54,55], which could have limited their findings.

The present study has various important strengths that reinforce the obtained results. Its strongest assets are the large sample size of CHD patients, the vast number of sociodemographic/lifestyle variables collected, and the inclusion of DI and HOMA-IR as co-variables. Moreover, this is a comprehensive dietary intervention with both healthy diets equally performed. Although dietary compliance could be a factor, in this case, adherence to the recommended dietary patterns was excellent, as shown by the rigorous dietary assessment measurements [59].

Our study also has certain limitations. First, our population included only CHD patients, which prevented us from generalizing the findings to other populations. Secondly, we used a FFQ to assess dietary exposure, which is known to contain measurement errors. However, we also used a validated FFQ and a standardized dietary assessment protocol to reduce possible information bias. Moreover, in both study groups, there were fewer women than men, although the proportion of both Incident-T2DM and non-T2DM was similar. Additionally, another limitation of the present study is that we did not include the measurement of physical activity.

## 5. Conclusions

The present study reports for the first time that changes in NRF9.3 (a DQI score) after one year of dietary intervention are related to the incidence of T2DM in the long term (5 years) in patients with CHD. In this case, both dietary interventions reported similar improvements in the NRF9.3 score. Patients in the lowest tertile of improvement of NRF9.3 had more than double the probability of developing T2DM after 5 years of dietary intervention. These results highlight the fact that the increase in diet quality, assessed as nutrient density, may be a risk predictor of T2DM onset in the following years. Thus, NRF9.3 could be a useful tool to identify and decrease the risk of T2DM in the long term of healthy dietary intervention.

## Figures and Tables

**Table 1:** Baseline clinical and metabolic characteristics, and lipid profiles of the study population.

Variables	Incident-T2DM	Non-T2DM	*p*-Value
n	106	316	
Men/Women (n)	86/20	266/50	0.466
Age (years)	58.8 ± 0.9	57.2 ± 0.5	0.127
Med diet/low-fat diet (n)	65/41	167/149	0.892
BMI (kg/m^2^)	31.4 ± 0.5	29.9 ± 0.2	0.001 *
Waist circumference (cm)	105.1 ± 1.1	101.4 ± 0.6	0.003 *
Triglycerides (mg/dL)	133.1 ± 6.7	117.9 ± 3.3	0.027 *
Total cholesterol (mg/dL)	165.3 ± 3.4	159.3 ± 1.6	0.090
HDL-cholesterol (mg/dL)	43.6 ± 1.1	44.3 ± 0.6	0.560
LDL-cholesterol (mg/dL)	93.5 ± 2.7	90.6 ± 1.4	0.313
HbA1c (%)	6.0 ± 0.03	5.8 ± 0.02	<0.001 *
Glucose (mg/dL)	96.2 ± 1.1	92.3 ± 0.6	0.001 *
Fasting insulin (mU/L)	10.4 ± 0.6	8.4 ± 0.3	0.005 *
ISI	3.4 ± 0.3	4.3 ± 0.2	0.001 *
IGI	0.66 ± 0.30	1.15 ± 0.08	0.023 *
HOMA-IR	3.36 ± 0.30	2.55 ± 0.09	0.001 *
Disposition index	0.77 ± 0.04	1.02 ± 0.03	<0.001 *
NRF9.3	724.6 ± 8.0	724.1 ± 5.4	0.964

Data expressed as mean ± standard error. Incident-T2DM: patients who developed T2DM. Non-T2DM: non-T2DM patients. T2DM, type 2 diabetes mellitus; Med diet, Mediterranean diet; BMI, body mass index; HDL, High-density lipoprotein; LDL, Low-density lipoprotein. HbA1c, glycated hemoglobin A1c; ISI, insulin sensitivity index; IGI, insulinogenic index; HOMA-IR: homeostatic model assessment; NRF9.3: Nutrient-Rich Food Index 9.3. One-way ANOVA *p*-values. * *p* < 0.05.

## Data Availability

Data described in the manuscript, code book and analytic code may be made available upon request after an accepted proposal for a scientific work due to privacy. Depending on the nature of the collaboration, electronic data, hard copy data, or biological samples may be required. All collaborations will proceed following the execution of a collaboration agreement. The terms of the collaboration agreement will be tailored to each specific collaboration, including the scope of shared documentation (such as de-identified participant data, data dictionary, biological samples, hard copies, or other specified datasets), which will be determined accordingly.

## Supplementary Materials

The following supporting information can be downloaded at: https://www.mdpi.com/article/10.3390/nu16081249/s1, Figure S1: Flow chart of the patients included in the analysis; Figure S2: 10-Step method for estimating added sugars content in food items described by Louie et al. [35]; Figure S3: Detailed 10-Step method for estimating added sugars content in food items in the CordioPrev Study; Supplementary Table S1: Alternative Healthy Eating Index 2010 (AHEI-2010) components and criteria for scoring; Figure S4: Effect of the dietary intervention on NRF9.3 score and diabetes status. Patients who became T2DM during the first year of intervention were excluded; Figure S5: Probability of T2DM development by COX analysis according to the tertiles of ΔNRF9.3. Patients who became T2DM during the first year of intervention were excluded.
