# Supplementary material for: Association between Diet Quality and Risk of Type 2 Diabetes Mellitus in Patients with Coronary Heart Disease: Findings from the CORDIOPREV Study
Source: Nutrients. 2024 Apr 22;16(8):1249. doi: 10.3390/nu16081249 (PMC11053861; doi:10.3390/nu16081249)
Supplement: Supplementary file 1 [file nutrients-16-01249-s001.zip › nutrients-2941703-supplementary.pdf]

Association between diet quality and risk of type 2 diabetes in patients with coronary disease: findings from the CORDIOPREV study

Lorenzo Rivas-García *et al*

SUPPLEMENTARY MATERIAL

Supplemental Figure 1S. Flow chart of the patients included in the analysis.

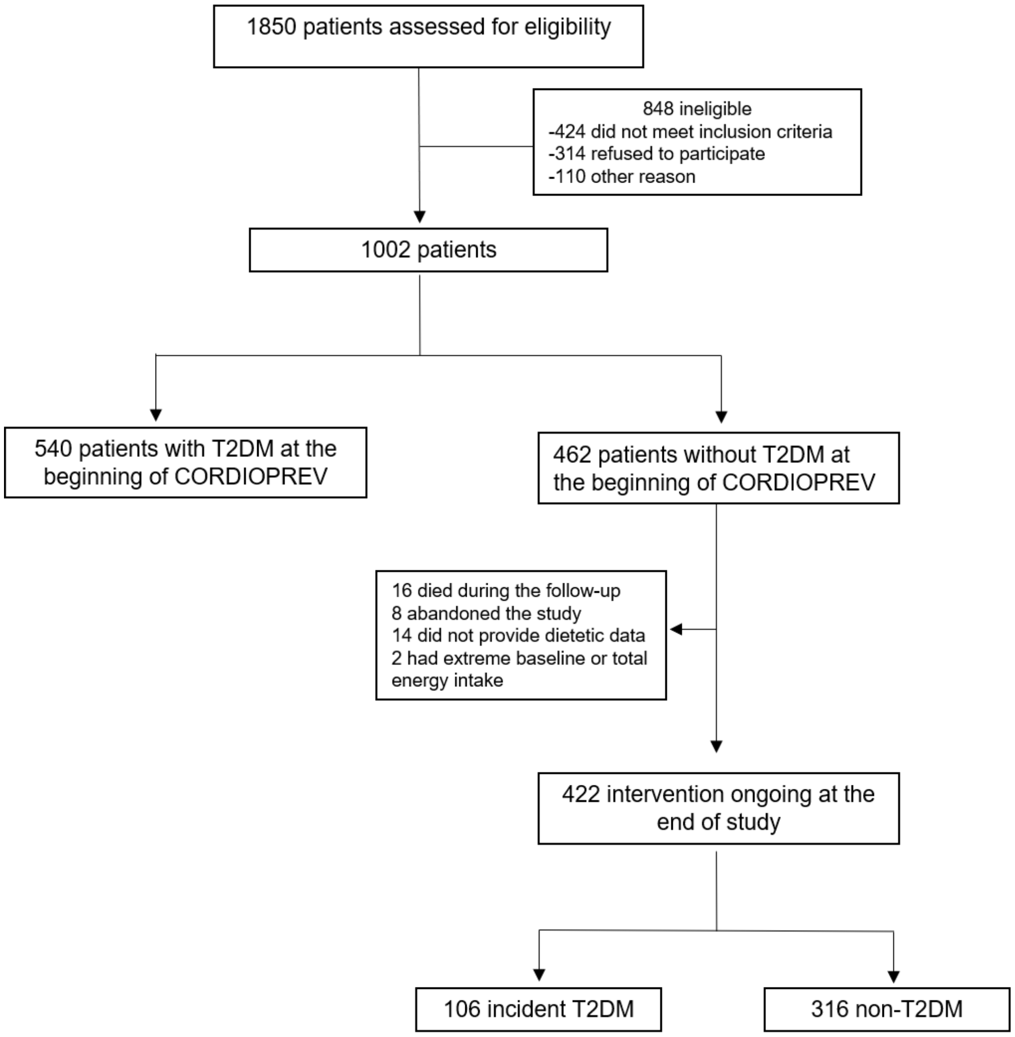

Association between diet quality and risk of type 2 diabetes in patients with coronary disease: findings from the CORDIOPREV study

Lorenzo Rivas-García *et al*

Supplemental Figure S2. 10-Step method for estimating added sugars content in food items described by *Louie* et al.

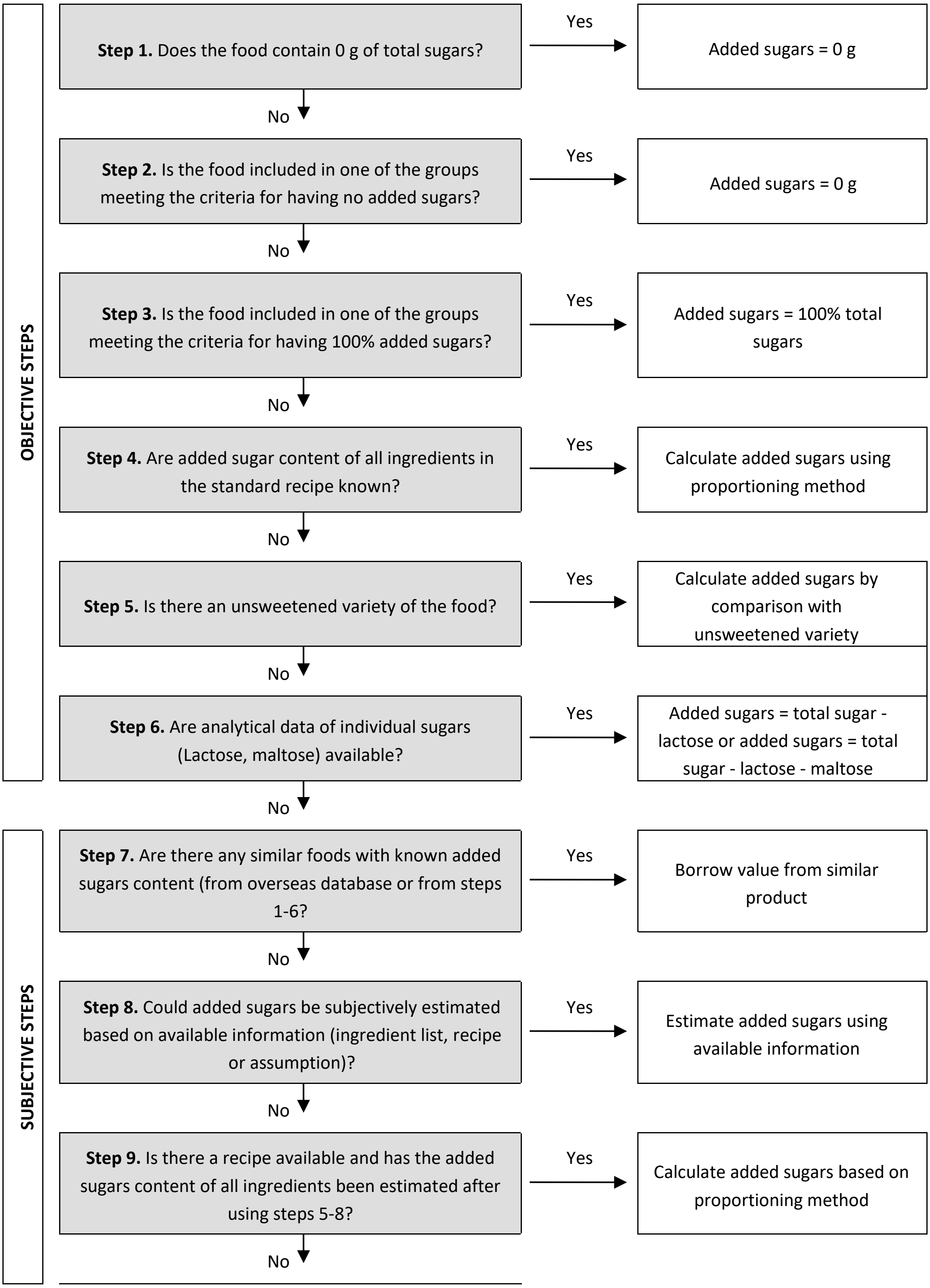

Association between diet quality and risk of type 2 diabetes in patients with coronary disease: findings from the CORDIOPREV study

Lorenzo Rivas-García *et al*

Step 10. Assign 50% of the total sugar as added sugars

Supplemental Figure S3. Detailed 10-Step method for estimating added sugars content in food items in the CordioPrev Study

Step 1. All foods with the total sugar content of 0g were assigned 0g of added sugars (n=30)

(1.a) Eggs (n=1)

(1.b) Fresh meat, liver, offal, cured ham (n=9)

(1.c) Fresh fish and seafood (n=8)

(1.d) Fats and oils (n=10)

(1.e) Salt (n=1)

(1.f) Tea (n=1)

Step 2. All foods unprocessed or minimally processed meeting the criteria for having no added sugars were assigned 0g of added sugars (n=54)

(2.a) Non-sugar-sweetened milk and dairy products (n=9)

(2.b) Fresh vegetables, potatoes and vegetable recipes (n=16)

(2.c) Fresh fruit, dried fruit and fresh fruit juices (without added sugar) (n=14)

(2.d) Nuts (n=2)

(2.e) Legumes (fresh and dried) (n=4)

(2.f) All plain cereal grains, pastas, rice and flours (n=4)

(2.g) All plain bread (n=2)

(2.h) All spices and herbs (n=3)

Step 3. Foods containing minimal intrinsic sugars were assigned 100% of total sugars as added sugar (n=33)

(3.a) Sugar, syrups and honey (n=2)<br>(3.b) Breakfast cereals (n=2)<br>(3.c) Savoury biscuits, sweet biscuits, donuts, muffins, cakes and pastries (n=10)<br>(3.d) Regular soft drinks (n=2)<br>(3.e) Instant coffee and decaffeinated (n=2)<br>(3.f) Processed meats (n=4)<br>(3.g) Non-sweetened alcoholic beverages (n=11)
Step 4. Calculation based on standard recipe used in the food composition database, where added sugar content of all ingredients was available from steps 1–3 (n=4)
Example- Added sugar content determined for canned fruits in syrup:<br>Added sugar (g/100g) = Total sugar (g/100g) – (total sugar content of raw fruit (g/100g) * %of raw fruit in the canned fruits = 21.5g – (9g*80/100) = 21.5g – 7.2g = 14.3g
Step 5. Calculation based on comparison with values from the unsweetened variety (n=0).
Step 6. Decision based on analytical data of lactose content (n=1).
Lactose subtracted from total sugars. Example – Sweetened low-fat yogurt: added sugar content (g/100g) calculated as total sugars (6.3g) – lactose (4.4g)
Step 7. Use "borrowed" values from similar products from steps 1–6 or from overseas databases (n=6)
Example – Milkshakes: added sugars estimated based on the added sugar content of *Nestle Nesquik Chocolate Milk*, 50% of the total sugars.
Step 8. Subjective estimation of added sugars based on the ingredient list of packed foods (n=3)
Example – Custard: added sugars estimated based on the ingredient list of *Danone Vanilla Custard* (80% milk, sugar, modified starch, stabilizers). 3.5g were considered as intrinsic sugars (lactose in milk) and deducted from the total sugars (11.8g)
Step 9. Calculation based on the standard recipe that includes ingredients with values assigned at steps 5–8, using the proportioning method (n=1).
Example – Homemade meatballs: ingredients ratio in the standard recipe checked (minced pork/beef meat, egg, garlic, breadcrumbs, parsley, virgin olive oil) and added sugars estimated as 0g

Step 10. Assign 50% of total sugars as added sugar (n=5).
Example – Precooked food products (e.g., ready-to-eat lasagne, croquettes or nuggets)

Association between diet quality and risk of type 2 diabetes in patients with coronary disease: findings from the CORDIOPREV study

Lorenzo Rivas-García *et al*

Supplemental Table S1. Alternative Healthy Eating Index 2010 (AHEI-2010) components and criteria for scoring

**Components of the AHEI-2010**	**Criteria for minimum score (0)**[a]	**Criteria for maximum score (10)**[a]
1. Vegetables [b]	0 servings/day	≥5 servings/day
2. Fruit [c]	0 servings/day	≥4 servings/day
3. Whole grains [d]		Women: 75 grams/day<br>Men: 90 grams/day
4. Sugar-sweetened beverages [e]	≥1 servings/day	0 servings/day
5. Nuts and legumes [f]	0 servings/day	≥1 servings/day
6. Red/processed meat [g]	≥1.5 servings/day	0 servings/day
7. *Trans* fats	≥4 % of energy	≤0.5 % of energy
8. Long-chain (n-3) fats (EPA+DHA) [h]	0 mg/day	250 mg/day
9. Polyunsaturated fats	≤2 % of energy	≥10 % of energy
10. Sodium [i]	Lowest decile	Highest decile
11. Alcohol [j]	Women: ≥2.5 drinks/day<br>Men: ≥3.5 drinks/day	Women: 0.5-1.5 drinks/day<br>Men: 0.5-2.0 drinks/day
Total score	0 points	110 points

[a] Intermediate intakes are scored proportionally between 0 and 10, except for whole grains.

[b] All types of vegetables, except potatoes. One serving = 0.5 cup of vegetables or 1 cup of green leafy vegetables (1 cup = 236.59g).

[c] Only fresh and whole fruit (not canned or dried fruit or juice). One serving = 1 medium piece of fruit or 0.5 cup of berries.

[d] Whole wheat bread, brown rice, whole pasta, and whole breakfast cereals. One serving of a 100% whole grain product (i.e., 0.5 cup of brown rice) = 15 to 20g of whole grains (per dry weight).

[e] Natural and commercial juices, regular and light cola, soda, and tonic water. One serving or drink = 200mL.

[f] Legumes include different types of beans, chickpeas, lentils, and peas. Nuts include walnuts, almonds, peanuts, hazelnuts, pistachios, and pine nuts. One serving of legumes = 0.5 cup. One serving of nuts = 30g.

[g] Red meats include pork, beef, cow, and lamb. Processed meats include cured ham, cooked ham, salami, sausages, chorizo, pork liver pate, hamburgers, meatballs, and bacon. One serving of red meat = 120g. One serving of processed meat = 45g.

[h] EPA, Eicosapentaenoic acid; DHA, Docosahexaenoic acid. The cut-off for optimal intake (250mg/day) can be achieved by consuming 2 servings of 120g of fish per week, with special attention to oily fish.

[i] Sodium intake is expressed in mg/day. The cut-offs for sodium are based on deciles of intake in the study population.

[j] No alcohol intake receives 2.5 points. One drink = 100mL of wine, 330mL of beer, or 50mL of liquor.

Association between diet quality and risk of type 2 diabetes in patients with coronary disease: findings from the CORDIOPREV study

Lorenzo Rivas-García *et al*

Supplemental Figure S4. Effect of the dietary intervention on NRF9.3 and diabetes status. Patients who became T2DM during the first year of intervention were excluded.

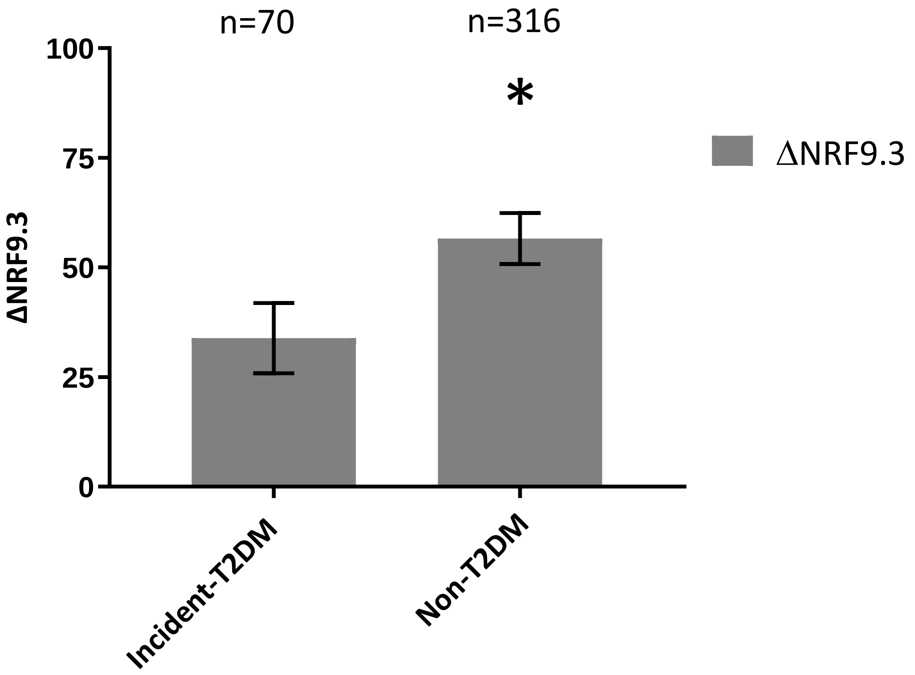

Data are presented as Δchanges of NRF9.3 produced between post- and pre-intervention ± SEM.

Variables were compared using the analysis of variance (univariate ANOVA) adjusted by age, sex, statin treatment, smoking habits, BMI, LDL, HDL, TG, HOMA-IR, ISI, DI and IGI.

Incident-T2DM (n = 70) and Non-T2DM (n = 316). Differences were considered to be significant when $p < 0.05$. *Significant differences between incident-T2DM and Non-T2DM.

Abbreviation: NRF9.3, Nutrient-Rich Food index 9.3; BMI, Body mass index; LDL, Low-density lipoprotein; HDL, high-density lipoprotein; TG, tryglicerides; HOMA-

IR, homeostatic model assessment; ISI, insulin sensitivity index; DI, disposition index; IGI, insulinogenic index.

Association between diet quality and risk of type 2 diabetes in patients with coronary disease: findings from the CORDIOPREV study

Lorenzo Rivas-García *et al*

Supplemental Figure S5. Probability of T2DM development by COX analysis according to the tertiles of ΔNRF9.3. Patients who became T2DM during the first year of intervention were excluded.

(A) unadjusted model; (B) adjusted model controlled for sex, statins, age, BMI, LDL, smoking habits, HDL, TG. C fully adjusted model controlled for sex, statins, age, BMI, LDL, smoking habits, HDL, TG., HOMA-IR, ISI, DI and IGI. Reference was the Tertile 1 (lowest). The hazard ratio (HR) between groups were calculated. Abbreviation: BMI, Body mass index; LDL, Low-density lipoprotein; HDL, high-density lipoprotein; TG, Tryglicerides¸ HOMA-IR, homeostatic model assessment; ISI, insulin sensitivity index; DI, disposition index; IGI, insulinogenic index.

A

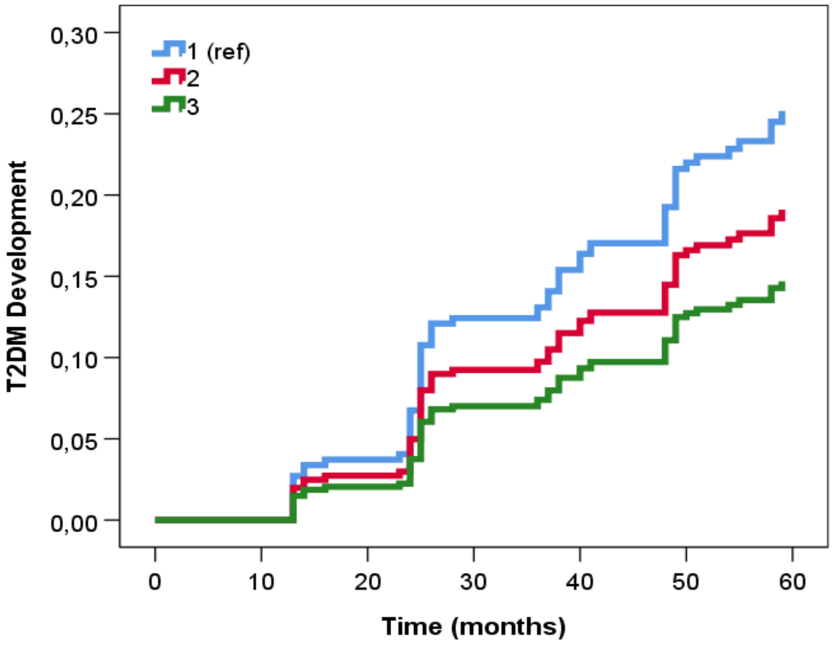

**Tertiles**	**HR**	**p-value**	**95% CI for HR**	
			Lower	Upper

**1 (ref)**	1.00		1.00	1.00
**2**	1.33	0.372	0.71	2.51
**3**	1.83	0.046*	1.01	3.30

B

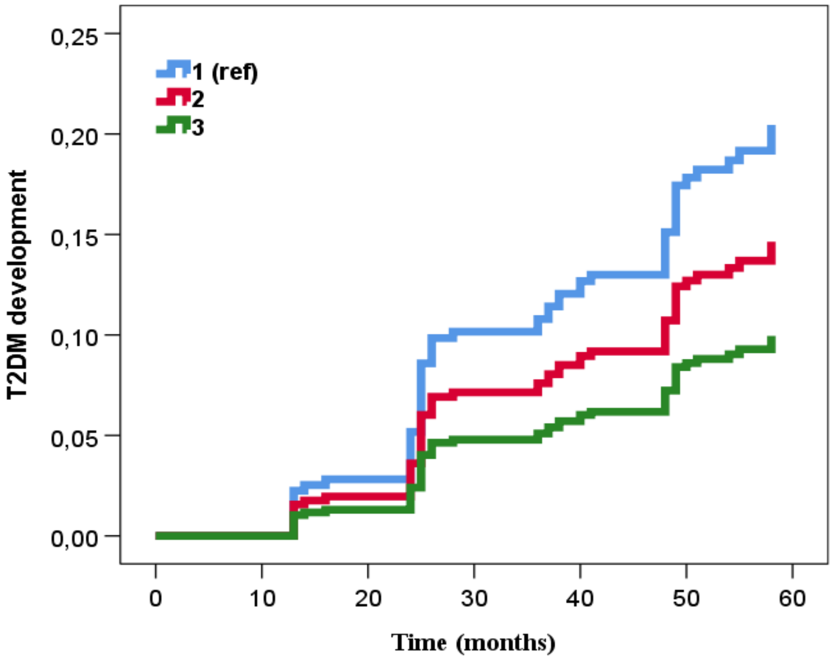

**Tertiles**	**HR**	**p-value**	**95% CI for HR**	
			Lower	Upper
**1 (ref)**	1.00		1.00	1.00
**2**	1.51	0.253	0.75	3.06
**3**	2.18	0.022*	1.12	4.27
